# Inhibition of hippocampal mossy fiber plasticity and episodic memory by human Aβ oligomers is prevented by enhancing cAMP signaling in Alzheimer's mice

**DOI:** 10.1002/alz.70194

**Published:** 2025-04-29

**Authors:** Shan‐Xue Jin, Jean‐Pierre Bellier, Adrienne Wells, Paula Montero LIopis, Praju Vikas Anekal, Jason S. Tresback, Barbara J. Caldarone, Lei Liu, Shaomin Li, Ulf Dettmer, Nagendran Ramalingam, Dennis J. Selkoe

**Affiliations:** ^1^ Ann Romney Center for Neurologic Diseases Department of Neurology Harvard Medical School and Brigham and Women's Hospital Boston Massachusetts USA; ^2^ MicRoN Core Harvard Medical School Boston Massachusetts USA; ^3^ Center for Nanoscale Systems Harvard University Cambridge Massachusetts USA; ^4^ Mouse Behavior Core Harvard Medical School Boston Massachusetts USA

**Keywords:** Alzheimer's disease, APP^NL‐G‐F^ knock‐in mice, Aβ oligomers, cAMP, episodic memory, FLIM‐FRET, long‐term potentiation, microgliosis, mossy fiber‐CA3 pathway, *β*‐adrenergic signaling

## Abstract

**INTRODUCTION:**

Early episodic memory impairment in Alzheimer's disease (AD) is linked to synaptic dysfunction from amyloid β‐protein oligomers (oAβ), particularly affecting the dentate gyrus mossy fiber‐CA3 pathway. The APP^NL‐G‐F^ mouse model exhibits early deficits in mossy fiber long‐term potentiation (mf‐LTP).

**METHODS:**

We administered the β‐adrenergic receptor agonist isoproterenol (ISO) in vivo and phosphodiesterase type 4 inhibitor GSK356278 in vitro to assess their impact on mf‐LTP and contextual fear memory. Fluorescence lifetime imaging (FLIM)‐Förster resonance energy transfer (FRET) microscopy was used to visualize impaired and rescued cyclic adenosine monophosphate (cAMP) signaling in dentate gyrus neurons.

**RESULTS:**

ISO prevented mf‐LTP impairment at 3–4 mo and improved memory by 7 mo. GSK356278 inhibited mf‐LTP deficits in a dose‐dependent manner. ISO also reduced hyperphosphorylation of synapsin I and microgliosis.

**DISCUSSION:**

These findings suggest that β‐AR activation and phosphodiesterase 4 (PDE4) inhibition mitigate oAβ‐induced memory deficits, supporting enhanced cAMP signaling as a therapeutic target for early AD.

**Highlights:**

- Early episodic memory deficits in AD linked to oAβ‐induced synaptic dysfunction.
- Isoproterenol and GSK356278 improve mossy fiber‐LTP and fear memory deficits.
- FLIM‐FRET shows treatments restore cAMP signaling in dentate gyrus neurons.
- Isoproterenol reduces synapsin I hyperphosphorylation and microgliosis.
- Enhancing cAMP signaling may help mitigate early memory deficits in AD.

## 1 BACKGROUND

Alzheimer's disease (AD) is characterized by a progressive decline in cognitive function, including disorientation, loss of memory, and impaired reasoning, leading to profound dementia and ultimately premature death. This highly prevalent neurodegenerative disorder is marked by extracellular deposits of diverse assemblies of amyloid β‐peptides (Aβ) as well as intraneuronal neurofibrillary tangles composed of the microtubule‐associated protein tau, associated with neuron loss.^1^ First‐generation transgenic amyloid precursor protein (APP) mouse models that overexpress human Aβ show inhibition of long‐term potentiation (LTP), an electrophysiological correlate of synaptic plasticity, and impaired learning and memory.^2^, ^3^ However, such APP mouse models not only overproduce Aβ but also overexpress APP and its non‐Aβ proteolytic fragments, complicating the biological interpretation of their phenotypes.^4^, ^5^ To address this issue, the Saito laboratory created several human mutant APP knock‐in mouse lines, including APP^NL‐G‐F^ mice, which produce high levels of human Aβ42 and elevated Aβ42/40 ratios in the brain without altering expression of endogenous APP and its other fragments.^5^

**RESEARCH IN CONTEXT**

1. **Systematic review**: The findings are positioned within the growing evidence highlighting the critical role of synaptic plasticity in cognitive decline associated with Alzheimer's disease (AD). Previous studies have identified various mechanisms by which amyloid β‐peptides (Aβ) oligomers impair neuronal signaling and cognitive functions. This investigation adds to the literature by focusing on the mossy fiber‐CA3 pathway in APP^NL‐G‐F^ mice, providing a model that better reflects human pathobiology compared to earlier transgenic mouse models.
2. **Interpretation**: The results suggest that the impairment of mossy fiber long‐term potentiation (mf‐LTP) due to low‐n Aβ oligomers can be effectively countered through β‐adrenergic receptor (β‐AR) activation and phosphodiesterase 4 (PDE4) inhibition. This aligns with established knowledge about the cyclic adenosine monophosphate/protein kinase A (cAMP/PKA) signaling pathway's involvement in synaptic plasticity. The demonstration that pharmacological interventions can lower synaptic dysfunction markers (like hyperphosphorylated synapsin I) and improve memory behaviors underscores the integral role of modulating neuroinflammatory responses—specifically, the reduction of microglial activation in AD progression.
3. **Future directions**: Future research should focus on longitudinal studies to evaluate the long‐term efficacy of β‐AR and PDE4 inhibitors on cognitive performance in various AD models. Additionally, the investigation of combined approaches involving immunotherapy targeting Aβ oligomers along with pharmacological strategies could yield synergistic effects in mitigating cognitive deficits. Exploring dose–response relationships and potential side effects of these therapeutic agents will also be essential for translating findings from animal models to clinical settings.

Aβ aggregates can interact with a variety of downstream targets,^1^, ^6^ activating different second‐messenger cascades that can alter neuronal plasticity, including LTP, learning, and memory.^7^ The hippocampus plays a central role in mammalian learning and memory, including via its classical trisynaptic circuit comprising the perforant pathway (the projections from the entorhinal islands to the dentate gyrus [DG]), the mossy fibers (mfs) (the projections from the DG to CA3), and the Schaffer collaterals (the projections from CA3 to CA1).^8^ Cyclic adenosine monophosphate (cAMP) is a crucial second messenger involved in both pre‐ and post‐synaptic plasticity across various neuronal types. In the hippocampal mf synapse, cAMP mediates presynaptic LTP and long‐term depression (LTD), primarily through the activation of protein kinase A (PKA).^9^

We recently demonstrated that presynaptic mf‐LTP is inhibited by applying diffusible Aβ oligomers (oAβ) isolated directly from AD *post mortem* brain and that this impairment can be prevented by selective activation of both β1 and β2 adrenergic receptors (ARs) and their downstream cAMP/PKA signaling pathway.^10^ While previous studies have shown the beneficial effects of cAMP/PKA signaling in AD models,^11^, ^12^, ^13^, ^14^ our study expands upon these findings by focusing specifically on the mf‐CA3 pathway, a synapse particularly vulnerable in early AD. Furthermore, unlike many prior studies that utilize APP overexpressing transgenic mice, we use the APP^NL‐G‐F^ knock‐in mouse model, which more faithfully replicates the gradual progression of Aβ pathology seen in human AD, without overexpression of APP.^5^ These results compellingly suggest the potential therapeutic application of drugs that enhance the cAMP/PKA signaling pathway in the treatment of AD. Here, we report that the in vivo administration of β1‐AR and β2‐AR agonists such as isoproterenol (ISO) to APP^NL‐G‐F^ mice via oral administration or else the in vitro application of the brain‐penetrant phosphodiesterase 4 (PDE4) inhibitor, GSK356278, to hippocampal slices of APP^NL‐G‐F^ mice, potently prevents human oAβ‐mediated impairment of mf‐LTP and ameliorates contextual fear memory deficits. Furthermore, we explore potential mechanisms by which ISO prevents mf‐LTP impairment in APP^NL‐G‐F^ mice. Finally, to extend these pathway‐specific findings, we utilize fluorescence lifetime imaging (FLIM)‐fluorescence resonance energy transfer (FRET) imaging in situ to investigate the dynamic impact of oAβ, particularly low‐n Aβ oligomers, on intracellular cAMP signaling.

## 2 METHODS

### 2.1 Mice

All experiments with mice were performed in accordance with the animal welfare guidelines of The Harvard Medical School and Brigham Women's Hospital.

The study utilized APP^NL‐G‐F^ mice (a gift from RIKEN Cen for Brain Science, Jan), C57BL/6J mice as a control, cAMPER mice (a gift from Dr. Kirill A. Martemyanov's Lab) and G32‐4 *Cre* mice (JAX006474). cAMPER;G32‐4 Cre mice were generated by crossing homozygous cAMPER females with hemizygous G32‐4 *Cre* males, as G32‐4 females may confer global *Cre‐*mediated deletion of *loxP*‐flanked sequences in some offspring. All mice were appropriately genotyped by Transnetyx (https://www.transnetyx.com), were healthy, and had not undergone any prior procedures. Mice of both sexes were used in this study. Because previous studies have reported no sex differences in amyloid pathology, inflammatory responses, and cognitive behavior,^15^, ^16^, ^17^ the data from male and female APP^NL‐G‐F^ mice were combined in our study. The mouse pups were weaned at 29 days of age and group‐housed (three–five mice per cage) in a controlled environment (22  ±  1°C, 50% humidity, and a 12‐h light:dark cycle) with free access to standard laboratory chow and water.

### 2.2 Hippocampal slice preparations and extracellular field recordings

Experiments were performed as previously described.^10^ Briefly, mice were anesthetized with halothane and decapitated. Transverse acute hippocampal slices (350 µm) were cut with a vibratome (Leica VT1000 S) in ice‐cold, oxygenated sucrose‐enhanced artificial cerebrospinal fluid (aCSF) containing 206 mM sucrose, 2 mM KCl, 2 mM MgSO_4_, 1.25 mM NaH_2_PO_4_, 1 mM CaCl_2_, 1 mM MgCl_2_, 26 mM NaHCO_3_, 10 mM D‐glucose, pH 7.4. After dissection, slices were incubated in aCSF that contained the following (in mM): 124 NaCl, 2 KCl, 2 MgSO_4_, 1.25 NaH_2_PO_4_, 2.5 CaCl_2_, 26 NaHCO_3_, 10 D‐glucose saturated with 95% O_2_ and 5% CO_2_ (pH 7.4), in which they were allowed to recover for at least 90 min before recording. Recordings were performed in the same solution at room temperature in a chamber submerged in aCSF. To record field excitatory postsynaptic potentials (fEPSPs) in the CA3 region of the hippocampus, standard procedures were used. A unipolar stimulating electrode (World Precision Instruments, Sarasota, FL, USA) was placed in the hilus region close to DG granule cell layer to stimulate mf axons. A borosilicate glass recording electrode filled with aCSF was positioned in stratum lucidum of CA3, 250‐350 µm from the stimulating electrode. AP5 (50 µM) was added in aCSF to prevent contamination with the NMDA receptor‐dependent pathway converging on CA3 neurons. Test stimuli were applied at low frequency (0.05 Hz) at a stimulus intensity that elicited a fEPSP amplitude that was 40%–50% of maximum and the test responses were recorded for 10 min before the experiment was begun to ensure stability of the response. To induce mf‐LTP, two consecutive trains (1 s) of stimuli at 100 Hz separated by 20 s were applied to the slices. Traces were obtained by pClamp 11 and analyzed using the Clampfit 11. Data analysis was as follows. The fEPSP magnitude was measured using the initial fEPSP slope and three consecutive slopes (1 min) were averaged and normalized to the mean value recorded 10 min before conditioning stimulus.

### 2.3 Western blots

For immunoblotting analysis, brains were rapidly dissected and chilled in ice‐cold phosphate buffered saline (PBS) 1X, then transferred to a cold surface to perform hippocampal dissections as described by Lein et al.^18^ Briefly, the hippocampus was first separated as an intact structure after peeling back the overlying cortex and was subdissected to yield areas CA3 tissues, which were immediately frozen on dry ice and stored at −80°C until use. CA3 Samples for electrophoresis were prepared by lysing cells in lysis tissues in RIPA buffer (50 mM Tris‐HCl pH 7.4, 150 mM NaCl, 1 mM EGTA, 1% NP‐40, 1% sodium deoxycholate, and 0.1% SDS) supplemented with protease and phosphatase inhibitors. 4X Laemmli buffer supplemented with 1.25% β‐mercaptoethanol was added to the lysate and boiled for 5 min. Samples were then electrophoresed on NuPAGE 4%–12% Bis‐Tris gels with NuPAGE MES‐SDS running buffer and SeeBlue Plus2 molecular weight marker (all from Invitrogen) at 140 V and transferred in the iBlot 2 system (Invitrogen) to nitrocellulose membranes (iBlot 2 NC regular stacks; IB23001). Membranes were fixed for 10 min in 0.4% paraformaldehyde (in PBS). Membranes were then blocked in blocking buffer (5% milk in Tris buffered saline with Tween‐20 [TBST]) for 1 h and incubated in primary antibody, phosphor‐Synapsin 1 (Ser9) from Cell Signaling technology or total Synapsin 1 antibodies from Synaptic Systems in blocking buffer overnight at 4 °C. Membranes were washed 5 × 5 min in TBST. Secondary antibodies were prepared in the blocking buffer and incubated for 1 h at RT. Membranes were washed 5 × 5 min in TBST and scanned (Odyssey CLx, Li‐Cor). All Western blots were processed in parallel and derived from the same experiment.

### 2.4 Immunofluorescence

Mice were sacrificed via isoflurane overdose inhalation and transcardially perfused with 0.1 M phosphate buffer (PB), followed by 4% paraformaldehyde (PFA) dissolved in 0.1 M PB. Brains were extracted and postfixed in 4% PFA for 24 h at 4°C, then transferred to 15% sucrose for an additional 24 h at 4°C before slicing 18 µm coronal sections through the extent of the hippocampus using a cryostat. The sections were blocked with 2% goat serum in 0.3% Triton X‐100 for 1 h at room temperature. Immunofluorescence was performed for double staining using a mouse anti‐amyloid β_17‐24_, 4G8 antibody (1:5000, Biolegend) in combination with a rabbit anti‐phosphor‐Synapsin 1 (S9) antibody (1:400, Biorbyt) or a rabbit anti‐glial fibrillary acidic protein (GFAP) antibody (astrocyte marker) (1:400, Invitrogen) or a rabbit anti‐Iba1 antibody (microglia marker) (1:200, Proteintech). Additionally, a mouse anti‐Iba1 antibody (1:200, Invitrogen) was used with either a rabbit anti‐β1‐AR antibody (1:200, Alomone labs) or a rabbit anti‐β2‐AR antibody (1:200, Alomone labs) and Sections were incubated overnight at 4°C. The following day, the sections were washed 3 × 5 min in PBS and then incubated for 1.5 h at room temperature with a mixture of Alexa 488 goat anti‐rabbit IgG (1:300, Invitrogen) and Alexa 647 goat anti‐mouse IgG (1:300, Invitrogen). Sections were subsequently washed 3 × 5 min in PBS, dried at RT and sealed with cover glass. The stained sections were examined with a Leica DMi8 fluorescence microscope and Image processing and analysis were done in FIJI ImageJ Version 2.1.0/1.54f. All imaging were acquired under a 20x, 0.4 NA objective with a resolution (1024 × 1024; pixel size  =  650 nm). Positive signals in each section were determined in 1024 × 1024 pixels areas of surrounding hilus between DG granule cell layer and CA3 pyramidal cell layer. For all quantitative analyses, the three most representative sections from each mouse were chosen, and labeled pS9 puncta, Aβ plaques, and cells in each section were counted. A customized FIJI ImageJ macro was developed to analyze acquired Phosphor‐Synapsin 1 (pS9) positive puncta data. The image is initially processed in FIJI ImageJ using Gaussian blur and rolling ball background subtraction. Next, a user‐selected threshold method is applied to create a puncta mask. The “Analyze Particles” function is then used with specified minimum and maximum size limits to quantify puncta number and size. Aβ plaques and Cells (astrocytes, microglial cells) were expressed as density (plaques/mm^2^ and cells/mm^2^).

### 2.5 Contextual fear conditioning

Contextual fear conditioning (CFC) test was employed to measure the amygdala‐and hippocampal‐dependent learning. CFC was conducted over two consecutive days, with a training phase on day 1 and a test phase on day 2. On day 1, mice were placed individually into the conditioning chamber (Med Associates, St Albans, VT, USA) and allowed to explore for 2 min before receiving 2, 2 s, 0.5 mA foot shocks, with an intertrial interval of 2 min. After the last foot shock, mice remained in the chamber for an additional 1 min before being returned to their home cages. On day 2, mice were placed back into the conditional chamber without receiving a shock for 5 min. Freezing responses were video recorded for a duration of 5 min, with Media Recorder 4 Software and analyzed with Noldus Ethovision Software (XT 17.5) (Leesburg, VA). CFC was assessed in 16 control APP^NL‐G‐F^ mice (6–7‐mo) receiving only plain water (10 males and 6 females) and 18 ISO‐treated APP ^NL‐G‐F^ mice (6–7‐mo) receiving ISO in their drinking water for 2 mo (10 males and 8 females). The percentage of freezing in the conditioning context on the test day served as an index of memory. All the chambers were cleaned with 70% ethanol before and after each test to eliminate any possible instinctive odor cues.

### 2.6 Aβ oligomer sample preparation and AFM experiments

Synthetic amyloid β_1‐42_ (human) was purchased from Anaspec Inc., (San Jose, CA, USA) and prepared for aggregation by resuspending lyophilized Amyloid β_1‐42_ in hexafluoroisopropanol (HFIP), followed by drying through vacuum lyophilization. The oAβs were generated according to the procedure described by Matsui et al.,^19^, ^20^ 0.12 mM solution of the monomer was prepared by dissolving the lyophilized peptide in a buffer solution consisting of 3.5% of 0.1 M NaOH in a 0.05 M phosphate buffer (pH 7.4) containing 0.05 M NaCl, and incubated for 3–6 h at 4°C. The resulting oAβ solution was diluted 200 times with Mili‐Q water, and 8 µL of this solution was dropped onto a freshly cleaved mica disc (V‐1 Grade 12 mm, Ted Pella, INC., Redding, CA, USA), then dried using a nitrogen stream. The AFM experiments were conducted in AC tapping mode in Air with the Asylum Research Cypher AFM (Oxford Instruments, UK) with a 240 AC cantilever (240AC‐NA, tip radius: < 7nm, spring constant: 2 N/m). The resonant frequency was approximately 70 kHz. Areas of 1 × 1 squared micrometer and 256 × 256 pixels were scanned at 1 Hz using Asylum Research software (version 16.31.232). The AFM images obtained were analyzed with Gwyddion software (v.2.65) and Asylum Research software (version 16.31.232) after masking and flattening the height images to eliminate scan artifacts and surface corrugation. The height values of Aβ observed in circular contrasts of AFM data were measured through cross‐sectional analysis. Several AFM images were acquired to generate the height histograms, which were subsequently subjected to Gaussian fitting analysis using Igor Pro 7 or 9.

### 2.7 FLIM and ratiometric FRET analysis

Hippocampal slices were prepared from 2‐ to 3‐mo‐old cAMPER;G32‐4 Cre mice, as described in the extracellular field recording section above. Slices were eventually transferred to a recording chamber that was continuously perfused with gas‐saturated aCSF at a flow rate of 2–3 mL/min. FLIM imaging (512 × 512) was performed using a fully motorized Leica Stellaris 8 DIVE FALCON upright multiphoton and confocal microscope outfitted with a Scientifica motorized stage and 4Tune spectral external detectors (NDDs). mTurquoise (FRET donor) was excited while simultaneously acquiring emission signals range of 465–505 nm (mTurquoise; FRET donor) and 526–578 nm (Venus; FRET acceptor). FRET donor excitation in acute brain slices was achieved by tuning a Spectra physics Insight X 3 dual beam at 850 nm with two‐photon imaging. The fluorescence images were acquired through a Plan Apo 25x, 1.0 NA Water dipping objective outfitted with an automated correction collar at approximately 1‐ to 2‐min intervals by selecting the minimize time interval possible between Z‐stack acquisition in the LasX acquisition software. Multiplane z‐stack (3 µm step size; 400 Hz scan speed; 2.0875 µs Pixel Dwell Time) were recorded to generate XYZt stacks for each time point. Lifetime information was acquired by setting the detectors to photon counting mode. FLIM, records the fluorescence decay of the donor, and a shorter fluorescence lifetime means more FRET.

Fluorescence intensity and the FRET signal were quantified within a region of interest (ROI) for individual neurons expressing TEpacVV, using a customized FIJI ImageJ macro as described that the DG neurons are segmented using an Otsu threshold algorithm on a difference of gaussian‐filtered donor image. To eliminate edge effects, the segmented DG neuron regions are eroded by two‐pixel widths. Before the ratio is taken of acceptor pixel intensity/donor pixel intensity within these segmented DG neuron regions, the background of both acceptor and donor images is subtracted using a rolling ball algorithm with radius of 50 pixels. The FRET signal was calculated as the ratio of acceptor emission Yellow Fluorescent Protein (YFP) to donor emission Cyan Fluorescent Protein (CFP), after subtracting the background fluorescence from both YFP and CFP signals. Various reagents were bath applied during the experiments.

### 2.8 Statistical analyses

All the resulting raw data were graphed, and statistical analyses were conducted using GraphPad Prism 10 software. Data are presented as mean ± SEMs or mean ± SD (only for Western blots data analysis). The specific statistical analysis performed is described in the legend of each figure. Significant differences were determined using either a one‐way analysis of variance (ANOVA) test with post hoc Tukey's test or an unpaired Student's *t*‐test, as appropriate.

## 3 RESULTS

### 3.1 mf‐LTP is impaired by age 3–4 mo in APP^NL‐G‐F^ mice, but this is prevented by 2‐mo pretreatment with oral isoproterenol

**FIGURE 1:**
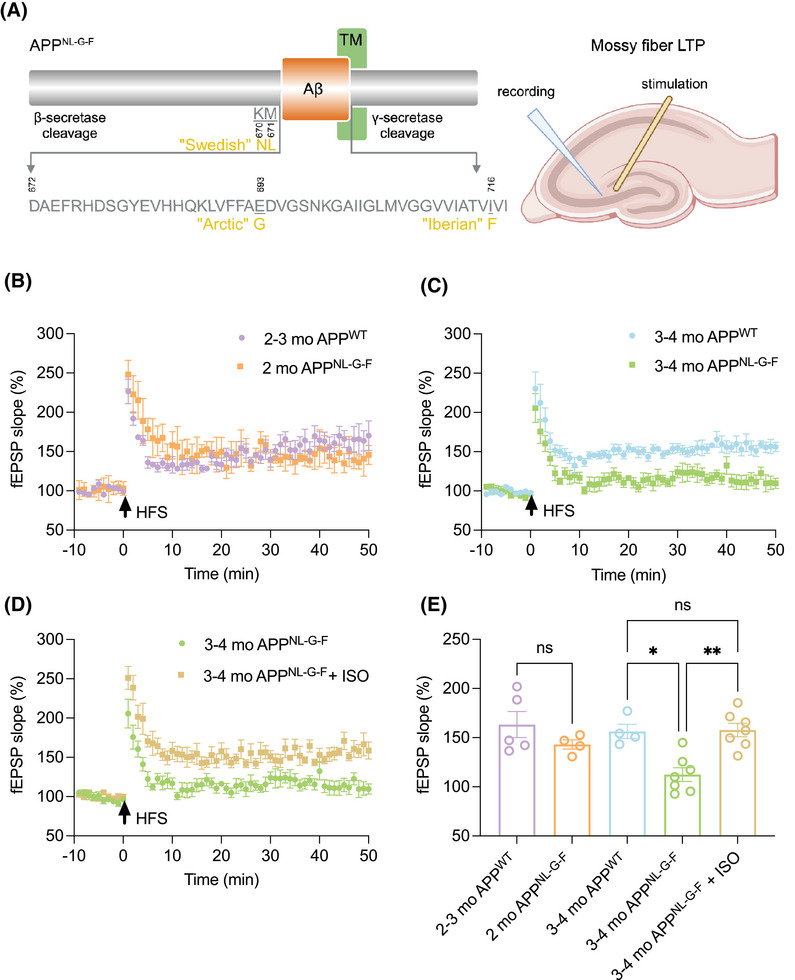
Deficits in mf‐LTP in APP^NL‐G‐F^ mice start at age 3–4 mo, but 2 mo pretreatment with oral isoproterenol prevents this. (A) Diagram of the APP and sequence of Aβ indicating Swedish, Arctic and Iberian mutations (left), as well as schematics of slice recording (right, created with BioRender.com). Time course LTP traces show that mf‐LTP is impaired in 3–4 mo APP^NL‐G‐F^ mice (C), but not in 2‐mo‐old APP ^NL‐G‐F^ mice (B), when compared to 3–4 mo wt mice. (D) Oral ISO prevented the defect in mf‐LTP in 3–4 mo APP^NL‐G‐F^ mice. (E) Histograms of the average potentiation for the last 10 min of mf‐LTP recordings. Each slice used for each experiment was from a different mouse. Error bars = means ± SEMs; *p*‐values calculated using one‐way ANOVA with Tukey's post‐test comparing the means of recorded fEPSP slopes. Compared to the same age in wt mice, mf‐LTP is impaired in 3–4 mo APP^NL‐G‐F^ mice (wt 156.3 ± 7.27 *n* = 4 vs. APP^NL‐G‐F^ 103.1 ± 8.32; *n* = 9, *p* = 0.004) but not in 2 mo APP^NL‐G‐F^ mice (wt 163.3 ± 13.32 *n* = 5 vs. APP^NL‐G‐F^ 143 ± 4.67; *n* = 4, *p* = 0.55). ISO prevented the decrease in mf‐LTP in 3 mo APP^NL‐G‐F^ mice (Ctrl: 103.1 ± 8.32 *n* = 9 vs. ISO: 157.8 ± 6.84 *n* = 7; *p* = 0.0005).

A major input to the hippocampus is provided by the DG.^21^ Information is transmitted to CA3 hippocampal neurons through mf synapses formed by the axons of dentate granule cells, which are believed to participate in the rapid encoding of contextual and episodic memories.^9^, ^22^, ^23^, ^24^, ^25^, ^26^ Episodic mnemonic processes are particularly affected early in AD and are the most common presenting symptom of the disease. Several studies have documented age‐dependent impairment in mf‐LTP in APP‐overexpressing transgenic mouse lines, including 12 mo old APP/PS1 mice and 24 mo old Tg2576 mice.^27^, ^28^ These transgenic mice express high levels of the human APP holoprotein and accumulate both Aβ and non‐Aβ proteolytic fragments of APP throughout life.^4^ In contrast, mice having AD‐causing human mutations knocked into the endogenous mouse APP locus bearing a humanized Aβ region (i.e., where the divergent moue Aβ residues [5, 10, and 13] have been changed to their human counterparts) accumulate high levels of human Aβ42 without changes in the expression of APP and its other constitutive fragments.^5^ The APP^NL‐G‐F^ mouse line was shown in the study by Saito et al. to exhibit age‐dependent Aβ amyloidosis, with cortical and hippocampal deposition beginning by 2 mo and nearly saturating by 7 mo.^5^ They observed normal LTP in the hippocampal CA1 region at 3–4 mo followed by deficits at 6–8 mo, as well as at 3–4 mo in prefrontal cortex (PFC),^29^ one of the first areas affected by amyloid pathology in humans.^30^ In the current study of APP^NL‐G‐F^ mice, we recorded LTP at the mf‐CA3 synapse, the second synapse in the hippocampal trisynaptic pathway, at ages 2 mo and 3–4‐mo. No significant differences in mf‐LTP were observed between APP^NL‐G‐F^ mice and wild‐type (wt) mice at age 2 mo (Figure 1B). However, mf‐LTP recordings at 3–4 mo showed a marked and statistically significant reduction in APP^NL‐G‐F^ compared to age‐matched wt mice (Figure 1C). Given that Aβ plaques are not prominent in the hippocampus of 3–4 mo old APP^NL‐G‐F^ mice (Figure S1), and that studies in other transgenic AD models (e.g., APP/PS1 mice) indicate that oAβ is the predominant Aβ species at this young age,^31^ we inferred that oAβ, rather than plaques, is primarily responsible for the observed mf‐LTP deficits. This interpretation is further supported by accumulating evidence that diffusible oAβ is more neurotoxic than fibrillar plaque Aβ.^32^, ^33^

In vitro (brain slice) studies have demonstrated that modulation of β‐ARs is a crucial upstream event for inducing mf‐LTP.^34^, ^35^ Furthermore, diffusible oligomeric Aβ isolated from the human (AD) cortex inhibits mf‐LTP in slices, and this can be prevented by activating β‐AR with the non‐selective agonist, ISO.^10^ Here, we sought to extend these findings to the in vivo context. We administered ISO in the drinking water at 0.1 g/L, as per Li et al.,^36^ to 5‐ to 6‐week‐old APP^NL‐G‐F^ mice for 2 mo. This treatment fully prevented the mf‐LTP impairment of 3–4 mo APP^NL‐G‐F^ mice (Figure 1D,E). These results indicate that deficits in mf‐LTP begin at age 3–4 mo in APP^NL‐G‐F^ knock‐in mice and that in vivo activation of the β‐ARs signaling pathway can prevent this impairment.

### 3.2 mf‐LTP inhibition can be dose‐dependently rescued by a PDE4 inhibitor in 3–4 or 6–7 mo APP^NL‐G‐F^ mice

It is well established that mf‐LTP requires activation of β‐ARs as well as downstream cAMP/PKA signaling cascades.^35^, ^37^, ^38^ Our previous studies demonstrated the benefits of activating of cAMP/PKA signaling using PDE4 inhibitors, which elevate cellular cAMP levels. This treatment can dose‐dependently prevent the inhibition of mf‐LTP by human oAβ added to hippocampal slices of wt mice perfused with oligomer‐rich AD brain aqueous (soaking) extracts.^10^

**FIGURE 2:**
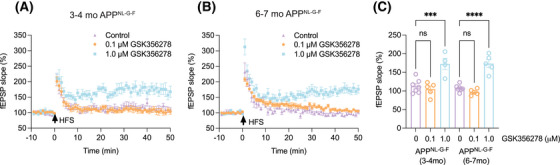
Dose‐dependence of PDE4 inhibitor GSK356278 rescue of mf‐LTP inhibition in 3–4 or 6–7 mo APP^NL‐G‐F^ mice. GSK356278 (1 µM but not 0.1 µM) prevented the inhibition of LTP in 3–4 mo (A, C 0 µM: 112.5 ± 6.98 *n* = 7 vs. 0.1 µM: 104.8 ± 8.15 *n* = 5, *p* = 0.978; 0 µM: 112.5 ± 6.98 *n* = 7 vs. 1 µM: 172.5 ± 15.09 *n* = 4, *p* = 0.0002) or 6–7 mo (B, C 0 µM: 107.3 ± 3.39 *n* = 7 vs. 0.1 µM: 96.98 ± 3.53 *n* = 5, *p* = 0.926; 0 µM: 107.3 ± 3.39 *n* = 7 vs. 1 µM: 173.3 ± 10.61 *n* = 5, *p* < 0.0001) APP^NL‐G‐F^ mice. Each slice used for each treatment was from a different mouse. Error bars = means ± SEMs. *p*‐Values calculated via one‐way ANOVA test; ***p < 0.001, ****p < 0.0001, ns, *p* > 0.05.

Here, we tested a newer brain‐penetrant PDE4 inhibitor, GSK356278, which potently reduces cAMP hydrolysis by PDE4B but has a modestly higher affinity for the high‐affinity rolipram binding site (HARBS) compared to the first‐generation PDE4 inhibitor, rolipram.^39^ We observed that human oAβ‐mediated mf‐LTP inhibition is prevented by rolipram at 3 µM (but not at 0.1 µM) when hippocampal slices from wt mice are exposed to AD brain soaking extracts mixed with rolipram (data not shown). We then incubated hippocampal slices from 3–4 mo APP^NL‐G‐F^ mice with 0.1 µM GSK356278 for 1 h followed by two trains of high frequency stimulation (HFS) to the slices and observed that this low dose had no effect on the oAβ‐mediated impairment of mf‐LTP. In contrast, incubation with 1 µM GSK356278 fully prevented the impairment of mf‐LTP by oAβ (Figure 2A,C). Consistent with these results, GSK356278 also demonstrated a dose‐dependent effect on the inhibition of mf‐LTP in 6–7 mo APP^NL‐G‐F^ mice (Figure 2B,C).

### 3.3 ISO prevents hyperphosphorylation of synapsin I at Serine9 in the CA3 region of 6–7 mo APP^NL‐G‐F^ mice

**FIGURE 3:**
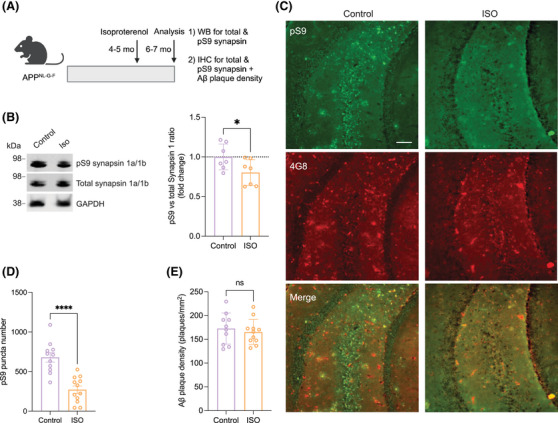
Isoproterenol prevents hyperphosphorylation of synapsin I at S9 in CA3 at 6–7 mo APP^NL‐G‐F^ mice. (A) Schematic depiction of ISO treatment protocol. (B) Western blots for total synapsin I and pS9. *n* = 7 pairs of animals. *, *p* < 0.05; mean ± SD. Paired student's t‐test. (C) Representative images of pS9 (green) and 4G8 (red) staining in hippocampal slices from control and ISO‐treated APP^NL‐G‐F^ mice. Scale bar: 100 µm. (D) and (E) are quantification of results (at least 10 slices from 4 mice each) from pS9 and 4G8 staining (C), respectively. Means ± SEMs. One way ANOVA test; ****p < 0.0001, ns, *p* > 0.05.

Synapsin I is a neuron‐specific phosphoprotein^40^, ^41^ and is localized to nerve terminals.^42^ It is associated mainly with synaptic vesicles and plays an important role in neurotransmitter release.^27^, ^43^ It serves as one of the major endogenous substrates for PKA and CaMKI, II, and IV.^44^ Numerous studies have indicated a role for Synapsin I in the presynaptic mechanisms of LTP and learning. Synapsin I may be a principal presynaptic effector for LTP triggered by the activation of the Ras/MAP kinase pathway following HFS.^45^, ^46^ Recent studies indicate that application of synthetic Aβ_1‐42_ aggregates to cultured rat hippocampal neurons induces hyperphosphorylation of synapsin I at serine9 (Ser9), and this form of phospho‐synapsin I is more abundant in cultured hippocampal neurons from the 5XFAD mouse model than its wt littermates.^47^ To investigate the potential effect of ISO on the phosphorylation of synapsin I at Ser9 in the mf‐CA3 pathway of APP^NL‐G‐F^ mice, we performed quantitative immunoblotting using antibodies against total synapsin I and Ser9 phosphorylation of synapsin I (pS9). In initial experiments, we found no significant difference in pS9 relative to total synapsin I among 3 mo wt mice, untreated (control) APP^NL‐G‐F^ mice, and orally ISO‐treated APP^NL‐G‐F^ mice (Figure S2). Therefore, we extended our study to 6–7 mo APP^NL‐G‐F^ mice, as cortical and hippocampal Aβ deposition is advanced by age 7 mo.^5^ In this older cohort, relative pS9 levels were significantly decreased in ISO‐treated versus untreated APP^NL‐G‐F^ mice (Figure 3B). To validate the findings by an independent method, we conducted co‐immunostaining with anti‐Aβ (4G8) and synapsin I pS9 antibodies in CA3 region of brain sections from 6–7 mo untreated and ISO‐treated APP^NL‐G‐F^ mice. Consistent with the immunoblotting data, the number of synaptic pS9+ puncta were markedly reduced in the ISO‐treated versus untreated APP^NL‐G‐F^ mice (Figure 3C,D). Importantly, the density of Aβ plaques did not differ significantly between the two groups (Figure 3C,E). Taken together, previous reports and our findings here suggest that ISO prevents mf‐LTP impairment in mature APP^NL‐G‐F^ mice in part by decreasing hyperphosphorylation of synapsin I at Ser9 in the CA3 region.

### 3.4 | ISO prevents microgliosis but not astrocytosis in CA3 of 6–7 mo APP^NL‐G‐F^ mice, and microgliosis contributes to oAβ‐mediated mf‐LTP impairment by releasing interleukin‐1β

**FIGURE 4:**
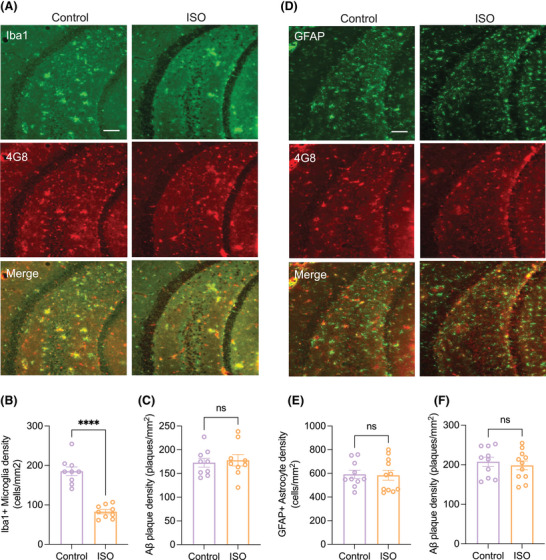
Isoproterenol prevents microglial but not astroglial inflammation in CA3 of 6–7 mo APP^NL‐G‐F^ mice. Representative images of Iba1 (green) and 4G8 (red) (A), as well as GFAP (green) and 4G8 (red) (D) staining in hippocampal slices from control and ISO‐treated APP^NL‐G‐F^ mice. Scale bar: 100 µm. (B) and (C) are quantification of results (at least 9 slices from 4 mice each) from Iba1 and 4G8 staining (A), respectively. (E) and (F) are quantification of results (at least 10 slices from 4 mice each) from GFAP and 4G8 staining (D), respectively. Means ± SEMs. One way ANOVA test; ****p < 0.0001, ns, *p* > 0.05.

Our previous work demonstrated the ability of ISO to prevent oAβ‐mediated microglia inflammation from intracerebroventricular (i.c.v) injection of oAβ‐rich AD brain extracts in wt mice.^48^ The APP^NL‐G‐F^ mouse line is characterized by occurrence of amyloid plaques, microgliosis, and astrocytosis.^5^ To assess whether ISO also prevents gliosis in CA3 of 6–7 mo APP^NL‐G‐F^ mice, we performed double immunostaining with antibodies 4G8, Iba1, or GFAP as markers of Aβ deposits, microglia, and astrocytes, respectively. Similar to the ISO effects we had reported with i.c.v injection of human oAβ‐rich extracts in wt mice,^48^ oral ISO treatment for 2 mo significantly diminished Iba1^+^ microglia density (Figure 4A,B) but did not affect GFAP^+^ astrocyte density (Figure 4D,E) in CA3 of 6–7 mo APP^NL‐G‐F^ mice.

**FIGURE 5:**
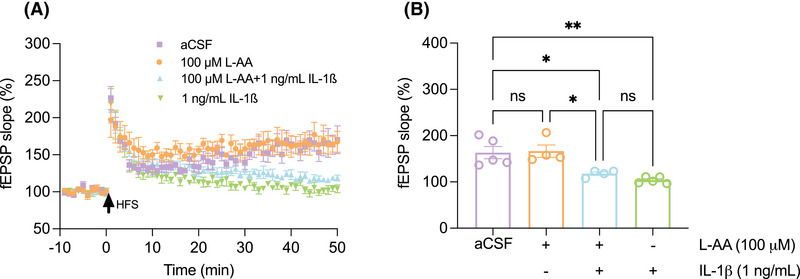
Microgliosis is involved in oAβ‐mediated mf‐LTP impairment in part by releasing IL‐1β. The astroglia toxin L‐AA (100 µM) (light yellow) does not block mf‐LTP (aCSF: 163.3 ± 13.32 *n* = 5 vs. L‐AA: 166.5 ± 13.44, *n* = 4, *p* = 0.99), but mf‐LTP was blocked by applying the pro‐inflammatory cytokine IL‐1β (1 ng/mL) alone (light green) (aCSF: 163.3 ± 13.32 *n* = 5 vs. IL‐1β: 103.7 ± 3.11, *n* = 5, *p* = 0.002) or 1 ng/mL IL‐1β and 100 µM L‐AA together (light blue) (aCSF: 163.3 ± 13.32 *n* = 5 vs. L‐AA+IL‐1β: 117.9 ± 3.68, *n* = 4, *p* = 0.03), when compared to aCSF control (light purple) (A), (B). Each slice used for each treatment was from a different mouse. Means ± SEMs. *p*‐Values used one‐way ANOVA with Tukey's post‐hoc test comparing the means of recorded fEPSP slopes.

    β‐ARs, specifically β1‐AR and β2‐AR, are mainly expressed in neurons but are also expressed on glial cells,^49^ particularly during activation of glial cells during stress and in certain disease conditions. To investigate the mechanism of ISO affecting microgliosis, we examined the expression of β1‐AR and β2‐AR in microglia in CA3 of 6–7 mo wt and APP^NL‐G‐F^ mice. We found that β1‐AR and β2‐AR were co‐localized on microglia in both wt and APP^NL‐G‐F^ mice (Figure S3A,B), suggesting that ISO treatment may have prevented microglial inflammation through β1‐ and β2‐ARs expressed on microglia. To prove the direct relationship between mf‐LTP and microgliosis, we next asked whether microgliosis is involved in the oAβ‐mediated impairment of mf‐LTP via the release of pro‐inflammatory cytokines such as interleukin (IL)‐1β or other potential mechanisms. IL‐1β is primarily expressed in microglia but also in astrocytes^50^ and has been implicated in regulating LTP in the hippocampus.^51^, ^52^, ^53^, ^54^ Given the established role of IL‐1β in modulating synaptic plasticity and its prominent release from activated microglia, we hypothesized that IL‐1β contributes to the oAβ‐mediated impairment of mf‐LTP. We first applied L‐α‐aminoadipate (L‐AA, 100 µM), which has been shown to be a gliotoxin specific for astrocytes,^55^ to hippocampal slices from wt mice. We found that mf‐LTP remained normal. Treatments with either IL‐1β alone or IL‐1β plus L‐AA together equally impaired mf‐LTP (Figure 5A,B). These results support that microgliosis is involved in part in oAβ‐mediated mf‐LTP impairment through release of IL‐1β in the APP^NL‐G‐F^ mice.

### 3.5 ISO ameliorates contextual fear memory deficits in 6–7 mo APP^NL‐G‐F^ mice

**FIGURE 6:**
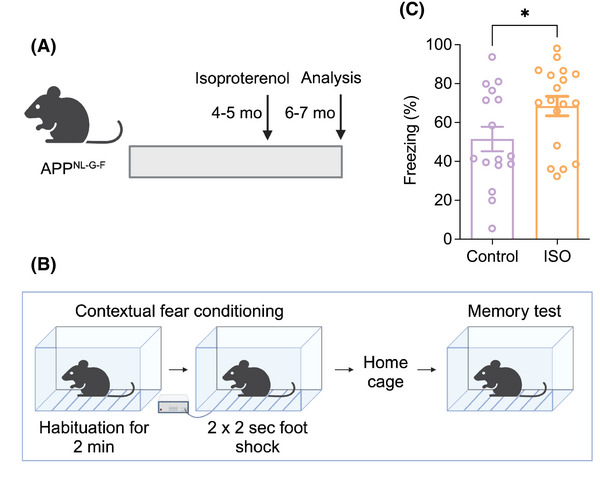
Isoproterenol ameliorates contextual fear memory impairment in 6–7 mo APP^NL‐G‐F^ mice. (A) Schematic depiction of ISO treatment protocol (B) Experimental design (C) Comparison of percentage of freezing of Control and ISO‐treated APP^NL‐G‐F^ mice during contextual fear tests (Control: 51.51 ± 6.31, *n* = 16 vs. ISO: 68.45 ± 5.02, *n* = 18, *p* = 0.04) Means ± SEMs. *p*‐Values used unpaired student's *t*‐test. *p < 0.05.

The mf‐CA3 synapse is widely recognized as required for the formation, storage, and retrieval of contextual memories in mammals.^26^, ^56^, ^57^ Previous studies have reported that APP^NL‐G‐F^ mice exhibit contextual fear memory deficits starting at age 6 mo.^17^ Therefore, we selected 34 male and female APP^NL‐G‐F^ mice aged 4–5 mo and divided them into two groups. One group, consisting of 16 mice (10 males and 6 females), received plain water for 2 mo as a control, while the other group, comprising 18 mice (10 males and 8 females), received the β‐AR agonist ISO in their drinking water for the same duration. After 2 mo, these now 6‐ to 7‐mo old APP^NL‐G‐F^ mice were subjected to a CFC test (Figure 6A,B). The ISO‐treated APP^NL‐G‐F^ mice exhibited a significantly higher percentage of freezing time compared to the control‐treated APP^NL‐G‐F^ mice (Figure 6C). Freezing was defined as the absence of all movement except for respiration for a minimum of 1 s. Freezing to the context are expressed as the percentage of observations in which freezing occurred. These findings suggest that ISO can ameliorate contextual fear memory deficits in 6–7 mo APP^NL‐G‐F^ mice.

### 3.6 Pure, synthetic oAβs decrease intracellular cAMP signaling; both ISO and GSK356278 rescue this as assessed by a FLIM‐FRET imaging paradigm

Previous findings using synthetic Aβ peptides and oAβ‐rich AD brain extracts indicated that low‐n Aβ oligomers may act as potent toxins for synaptic plasticity.^58^, ^59^, ^60^, ^61^, ^62^ mf‐LTP has been shown to be mediated by β‐AR/cAMP/PKA signaling pathways.^35^, ^37^, ^38^ To assess whether Aβ oligomers directly affect the downstream cAMP signaling, we performed real‐time monitoring of endogenous cAMP dynamics using FRET imaging in the cAMPER‐CA3 mouse model, which involved crossing cAMP sensor encoded reporter (cAMPER) mice^63^ with Cre‐CA3 mice to express the cAMP sensor selectively and conditionally in DG and CA3 of the hippocampus. Here, we first employed a protocol for synthetic Aβ oligomerization,^19^, ^20^ designed to predominantly generate low‐n Aβ42 oligomeric assemblies from pure Aβ42 monomers. We then probed this preparation by atomic force microscopy (AFM). This revealed that a freshly prepared solution of synthetic Aβ42 monomers results in a single Gaussian peak corresponding to an average size of 0.6 ± 0.18 nm (Figure S4A). After incubating the solution for 3–6 h at 4°C, three Gaussian peaks with average size of 0.98 ± 0.16, 1.8 ± 0.32 and 2.38 ± 0.41 nm are observed (Figure S4B). Based on previous AFM analyses,^19^, ^20^, ^64^ this represents a mixture of dimer, trimer and tetramer (peak 1), pentamer (peak 2), and octamer (peak 3), respectively. In contrast, two Gaussian peaks at 0.66 ± 0.13 and 1.18 ± 0.07 nm were observed from a freshly prepared solution of synthetic Aβ37 monomers (known to resist oligomerization compared to Aβ42) under the same incubation condition, indicating the presence of predominantly monomers and lower oligomers of Aβ37 (Figure S4C).

**FIGURE 7:**
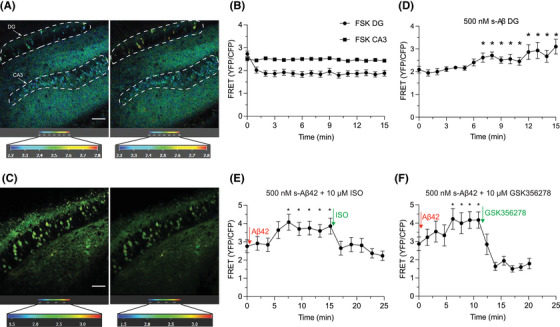
Pure synthetic low‐n Aβ oligomers can decrease intracellular cAMP signaling, and ISO or GSK356278 each rescue it as measured by endogenous FLIM‐FRET imaging. (A) Pseudocolor FLIM images before (left side) and after 1 min bath application of FSK (25 µM) (right side) to hippocampal brain slices. Scale bar = 50 µm and color bar = 2.2‐2.8 ns. (B) Time course of averaged FRET ratio recorded in DG granule cells and CA3 pyramidal neurons shown in (A) after stimulation with 25 µM FSK (*n* > 10 neurons for DG and CA3, respectively) (C) Pseudocolor FLIM images before (left side) and after 7 min bath application of synthetic oligomeric Aβ42 (500 nM) (right side) to hippocampal slices. Scale bar = 50 µm and color bar = 1.4–3.4 ns. (D) Time course of averaged FRET ratio recorded in dentate granule cells shown in (C) after stimulation with 500 nM synthetic oligomeric Aβ42 (*n* > 10 neurons. Both 10 µM ISO and 10 µM GSK356278 each rescue a slow increase in the FRET signal in the DG granule cells 7.6 min and 6.2 min after bath application of 500 nM synthetic oligomeric Aβ42 (E), (F), respectively. *n* > 10 neurons for each group. Means ± SEMs. One way ANOVA test; *p < 0.05.

We next examined the intracellular cAMP dynamics of DG and CA3 neurons expressing the FRET‐based cAMP sensor TEpacVV^63^ (the cAMP‐binding protein Epac1, sandwiched between suitable donor‐ and acceptor fluorescent proteins) by pharmacologically stimulating them through FLIM‐FRET. FLIM, records the fluorescence decay of the donor, and a shorter fluorescence lifetime means more FRET. We initially tested the efficacy of the system by stimulating the neurons with a positive control: the adenylyl cyclase activator forskolin (FSK, 25 µM), which increases intracellular cAMP levels. We found that the FRET acceptor (YFP) to donor (CFP) ratio decreased in the DG granule neurons but not in CA3 pyramidal neurons (Figure 7A,B). This observation is consistent with the characteristics of the FRET‐based cAMP sensor, which indicates an increase in the intracellular cAMP concentration as a decline in the FRET YFP to CFP ratio.^63^, ^65^ To further investigate whether intracellular cAMP levels in DG neurons are decreased by Aβ oligomers, we added low‐n Aβ42 oligomers (500 nM) to hippocampal brain slices. This treatment showed an increase in the FRET ratio in DG cells, with a definite change observed at a mean of 7.8 ± 0.58 min (*n* = 5 slices) after bath application of the Aβ42 oligomers (Figure 7C,D). However, the FRET ratio in DG neurons no longer rose significantly when our oligomer‐preferring Aβ antibodies 71A1 (2.12 µg/mL) or hB28 (3.01 µg/mL) were added to the brain slices perfusate with the 500 nM low‐n Aβ42 oligomers. As a negative control, the addition of 500 nM synthetic Aβ37 monomers alone (which oligomerize very little) also did not increase the FRET ratio (Figure S5A–C). Taken together, these results indicate that oAβs reduce the endogenous cAMP concentration (i.e, raise the FRET YFP:CFP ratio) in DG granule neurons, whereas the two oAβ‐neutralizing antibodies prevent this effect, and the non‐aggregating Aβ37 monomers do not alter cAMP concentrations.

Finally, to further establish whether the decrease in intracellular cAMP signaling caused by low‐n Aβ42 oligomers can be reversed through the activation of the β‐AR‐cAMP‐PKA signaling pathway, we applied ISO (10 µM) or GSK356278 (10 µM) to the brain slices of the cAMPER‐CA3 mice. Consistent with our electrophysiological findings (Figure 1, 2), we observed that both ISO and GSK356278 each prevented the slow decrease in intracellular cAMP signaling in DG granule neurons caused by the synthetic Aβ42 oligomers (Figure 7E,F).

## 4 DISCUSSION

**FIGURE 8:**
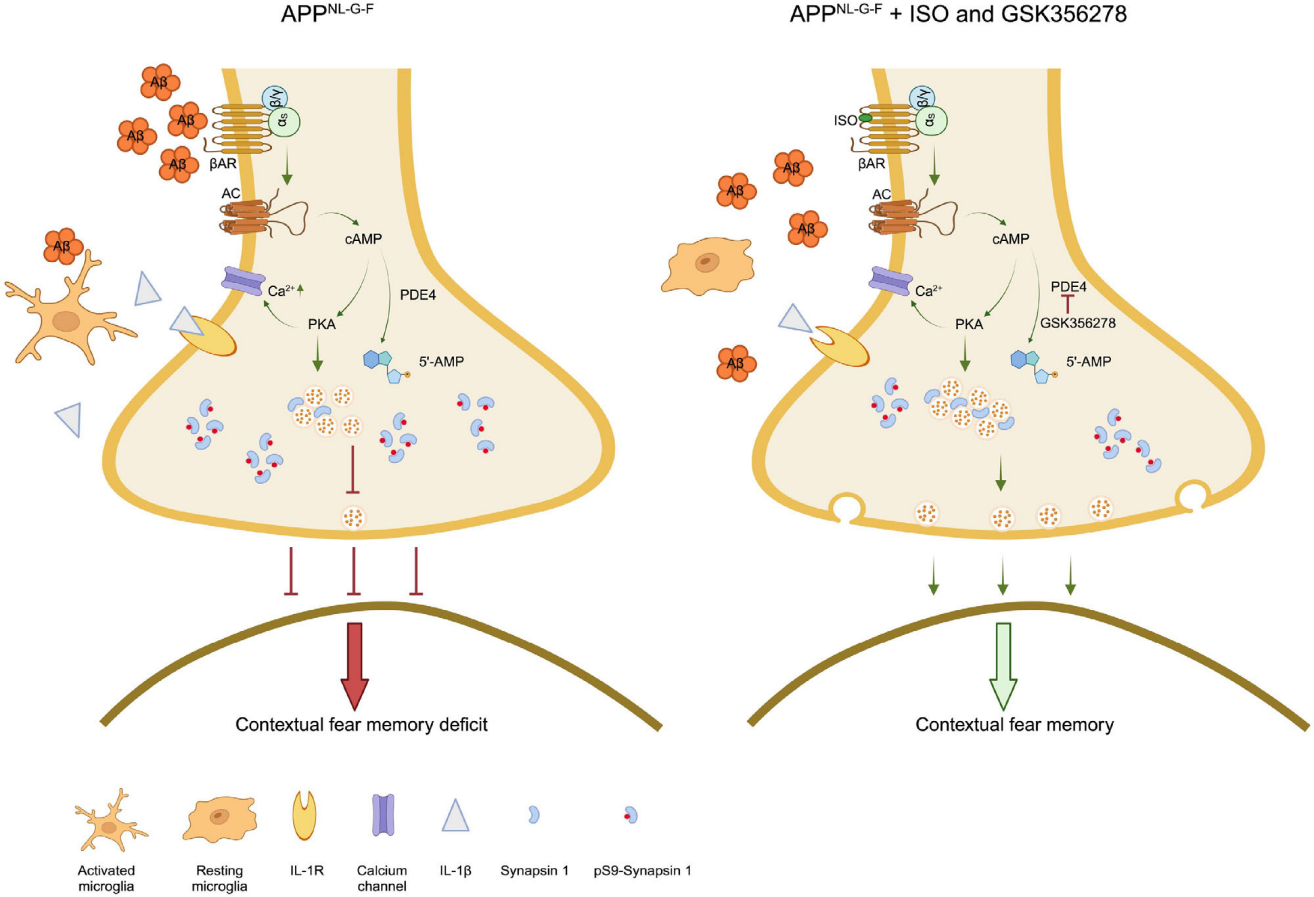
Model of the proposed mechanism of ISO and GSK356278 protection against Aβ‐induced synaptic dysfunction and cognitive impairment. oAβ triggers a cascade of detrimental events leading to synaptic plasticity deficits and cognitive decline. This cascade includes: microglial activation/IL‐1β release; hyperphosphorylation of synapsin I; and disruption of β‐AR/cAMP/PKA signaling. These events contribute to impaired mf‐LTP and deficits in contextual fear memory. Both ISO and GSK356278 offer protection against these Aβ‐induced impairments by targeting distinct points in this cascade. ISO restores cAMP/PKA signaling, which in turn normalizes PKA‐mediated phosphorylation of synapsin I. In addition, ISO mitigates microglial activation, thereby reducing IL‐1β release. GSK356278, a PDE4 inhibitor, also elevates cAMP levels and promotes appropriate synapsin I phosphorylation. By restoring cAMP signaling and normal PKA‐mediated phosphorylation of Synapsin I, before Aβ accumulation reach a critical threshold to prevent subsequent oAβ‐induced aberrant synapsin I phosphrylation, as well as ISO mitigates microglial activation, ultimately preventing mf‐LTP deficits and improving contextual fear memory.

This study establishes a key role for β‐AR‐cAMP‐PKA signaling in the response of the DG mf circuit to soluble human Aβ oligomers, using in vivo and in vitro approaches. We explore the therapeutic effects of β1‐AR/β2‐AR activation (ISO) and PDE4 inhibition (GSK356278) on mf‐LTP and contextual fear memory in APP^NL‐G‐F^ knock‐in mice, revealing potential mechanisms and effects of low‐n human Aβ42 oligomers on intracellular cAMP signaling. This is the first demonstration that diffusible Aβ42 oligomers significantly impair cAMP signaling in DG granule neurons, potentially correlating with early AD deficits in synaptic plasticity and episodic memory. FLIM‐FRET in situ imaging provided visualization of endogenous cAMP responses. While Vitolo et al.,^11^ showed that enhancing cAMP signaling can prevent Aβ‐induced synaptic impairments, our study uniquely demonstrates that this protection specifically targets mf‐LTP, a synapse critical for episodic memory. We also identify potential underlying mechanisms, including the reduction of synapsin I hyperphosphorylation and the prevention of microgliosis (Figure 8), offering novel therapeutic insights.

We observed that ISO treatment prevents the hyperphosphorylation of synapsin I at Ser9 in the CA3 region of 6‐ to 7‐mo old APP^NL‐G‐F^ mice accumulating both soluble and insoluble Aβ deposits. This is significant given the well‐established role of synapsin I in synaptic function and plasticity, particularly in mf‐LTP. Previous studies have linked hyperphosphorylation of synapsin I at Ser9 to synaptic dysfunction in AD, with increased levels of phosphorylated synapsin I observed in response to Aβ exposure.^47^

While ISO treatment did not affect pS9 levels in young (3 mo) APP^NL‐G‐F^ mice, it significantly reduced pS9 levels in the middle‐aged (6–7 mo) cohort. This suggests that the pathobiological changes associated with oAβ accumulation reach a critical threshold by 6–7 mo of age, leading to synaptic alterations that can be mitigated by oral ISO treatment. The lack of significant differences in amyloid plaque density between the ISO‐treated and control mice further supports the idea that ISO's benefit is likely mediated through the influence of soluble oAβs on synapsin I phosphorylation rather than direct effects on insoluble amyloid plaque burden.

Our co‐immunostaining experiments corroborated the immunoblotting data, showing a reduction in synaptic pS9 puncta following ISO treatment. This supports the idea that ISO can restore synaptic integrity by preventing oAβ‐induced hyperphosphorylation of synapsin I. This further aligns with the hypothesis that synapsin I acts as a critical presynaptic effector in the Ras/MAP kinase pathway, activated during high‐frequency stimulation and essential for LTP.^45^, ^46^ By preventing the hyperphosphorylation of synapsin I, ISO could help preserve the mechanisms underlying mf‐LTP, potentially contributing to synaptic resilience in the context of Alzheimer's pathology.^66^, ^67^ The observed reduction in synapsin I hyperphosphorylation following ISO treatment suggests that PKA, a key downstream target of cAMP, plays a critical role in mediating the protective effects of β‐adrenergic signaling. PKA‐mediated phosphorylation of synapsin I at Ser9 is known to regulate synaptic vesicle trafficking and neurotransmitter release.^68^ It is possible that ISO treatment is restoring normal PKA‐mediated phosphorylation of synapsin I, thereby improving synaptic function. In addition to synapsin I, PKA is known to phosphorylate a variety of other synaptic proteins, including AMPA receptors and CREB.^69^, ^70^, ^71^, ^72^ Thus, the beneficial effects of ISO on mf‐LTP and contextual fear memory we demonstrate could be mediated at least in part by PKA‐dependent phosphorylation of these synaptic proteins. Future studies could investigate the effects of ISO and GSK356278 on the phosphorylation of these other PKA targets in the hippocampus of APP^NL‐G‐F^ mice.

Additionally, in APP^NL‐G‐F^ mice, an increase in the density of microglia in hippocampus was observed at 28 weeks (6–7 mo), but not in the density of astrocytes.^73^ Consistent with the report, we observed an increase in microglial density in the hippocampus of 6‐ to 7‐mo‐old APP^NL‐G‐F^ mice, while astrocyte density remained unchanged. This underscores the role of microglial cells in AD neuroinflammatory. Microglia activation contributes to synaptic dysfunction and cognitive decline.^5^, ^74^ Our results align with our earlier report indicating that ISO can mitigate microglial inflammation induced by oligomeric Aβ in vivo,^48^ suggesting a consistent protective mechanism.

The observed reduction in activated microglia density following ISO treatment may be mediated through the activation of the β1‐ and β2‐ARs which are expressed on microglia. This finding is significant as it highlights a potential pathway through which ISO exerts its anti‐inflammatory effects. The co‐localization of β1‐AR and β2‐AR with microglia in both wt and APP^NL‐G‐F^ mice suggests that these receptors may play a role in modulating microglial responses to gradual Aβ accumulation. Therefore, ISO‐mediated activation of microglial β‐ARs could dampen oAβ‐initiated sustained neuroinflammation, preventing the detrimental effects of prolonged microglial activation on synaptic function and plasticity.^74^, ^75^

The observation that IL‐1β alone impaired mf‐LTP, coupled with the finding that ISO treatment prevented microgliosis, strengthens the argument for a causal link between microglial activation and synaptic dysfunction in APP^NL‐G‐F^ mice. While we cannot rule out the possibility that microglial activation is secondary to oAβ accumulation, our results suggest that activated microglia contribute to the oAβ‐induced impairment of mf‐LTP through the release of pro‐inflammatory cytokines such as IL‐1β. Previous studies have shown that activated microglia can directly impair synaptic function through several mechanisms, including the release of reactive oxygen species (ROS) and the phagocytosis of synapses.^76^, ^77^ It is possible that ISO treatment is preventing these detrimental effects of microglia by reducing their activation state, thereby limiting the release of IL‐1β and other factors such as tumor necrosis factor‐alpha (TNF‐α)^78^ that impair synaptic function.

Our findings demonstrate that ISO treatment significantly ameliorated contextual fear memory deficits in 6–7 mo APP^NL‐G‐F^ mice. This is noteworthy given the established role of the mf‐CA3 synapses, the second synapses in the entorhinal cortex‐hippocampal (EC‐HPC) circuit, in the formation, storage, and retrieval of contextual memories,^26^, ^56^, ^57^ although it is important to acknowledge that the CA3‐CA1 synapses (third synapses), also play a significant role.^79^ This suggests that, while ISO may be influencing synaptic plasticity at the mf‐CA3 synapse, its ultimate impact on contextual fear memory likely involves a more complex interplay with downstream effects on the CA3‐CA1 pathway. Other relevant brain regions, such as coordinated activity between the hippocampus and amygdala, particularly the ventral CA1 to amygdala pathway, is known to be important for contextual fear learning.^80^, ^81^ It is possible that ISO treatment could indirectly influence this pathway, either through its effects on CA1 activity or through other mechanisms. Furthermore, the fact that CA3‐CA1 LTP is impaired in 6‐ to 8‐mo‐old APP^NL‐G‐F^ mice^29^ complicates our interpretation. While this impairment might suggest a reduced capacity for CA3‐CA1‐dependent memory processes, it's possible that ISO treatment is partially compensating for this deficit by enhancing synaptic plasticity at the mf‐CA3 synapse and/or through indirect effects on other pathways. Alternatively, the observed improvement in contextual fear memory despite impaired CA3‐CA1 LTP could suggest that the mf‐CA3 synapse plays a more dominant role in the specific contextual fear memory task we employed in APP^NL‐G‐F^ mice, especially considering the early stage of AD pathology in these mice. Future studies using more specific interventions targeting different hippocampal subregions are warranted to fully elucidate the relative contributions of these pathways. APP^NL‐G‐F^ mice exhibit contextual fear memory impairments as early as 6 mo.^17^ The improvement in freezing behavior in ISO‐treated AD mice suggests that β‐ARs activation enhances contextual and episodic memory.

We observed that low‐n Aβ42 oligomers significantly decreased intracellular cAMP levels in DG granule cells. This is concerning given the crucial role of β‐AR‐cAMP‐PKA signaling in mf‐LTP and memory.^35^, ^37^, ^38^ Understanding how oAβs impair cAMP signaling could identify therapeutic targets. Importantly, both ISO and GSK356278 restored cAMP signaling in the presence of Aβ42 oligomers. Moreover, oligomer‐preferring Aβ antibodies protected against the cAMP‐reducing effects of diffusible Aβ42 oligomers, further supporting the therapeutic potential of targeting these species. This underscores immunotherapeutic strategies in AD, as neutralizing Aβ oligomers may not only prevent synaptic dysfunction but also preserve cognitive function.^82^, ^83^, ^84^ The lack of substantial Aβ37 monomer aggregation further emphasizes the specificity of low‐n Aβ42 oligomers in disrupting cAMP signaling and supports the current development of γ‐secretase modulators to enhance the presenilin‐mediated processive processing of APP from longer to shorter Aβ peptides.^85^

FLIM‐FRET imaging visualized the detrimental effects of Aβ42 oligomers on cAMP signaling in live neurons. The ability of ISO and GSK356278 to enhance cAMP signaling in situ presents a promising strategy for addressing cognitive deficits associated with AD. Recent studies have shown that cAMP stimulation can promote proteasome activity, leading to the clearance of misfolded proteins, including tau.^86^, ^87^ It is possible that the beneficial effects of ISO and GSK356278 observed in our study are also mediated, at least in part, by enhanced proteasome activity, leading to the clearance of oAβs or other toxic proteins in the synapse. Future studies could investigate the effects of these interventions on proteasome activity in the hippocampus of APP^NL‐G‐F^ mice.

## CONFLICT OF INTEREST STATEMENT

D.J.S. is a director of Prothena Biosciences and ad hoc consultant to Eisai and Roche. The other authors declare no competing interests. Author disclosures are available in the supporting information.

## CONSENT STATEMENT

We confirmed that no human subjects participated in this study.

## Supporting information



Supporting Information

Supporting Information

Supporting Information

Supporting Information

Supporting Information

Supporting Information
