# Supplementary material for: Inhibition of hippocampal mossy fiber plasticity and episodic memory by human Aβ oligomers is prevented by enhancing cAMP signaling in Alzheimer's mice
Source: Alzheimers Dement. 2025 Apr 29;21(4):e70194. doi: 10.1002/alz.70194 (PMC12040739; doi:10.1002/alz.70194)
Supplement: Supplementary file 1 — Supporting Information [file ALZ-21-e70194-s005.pdf]

FIGURE S1

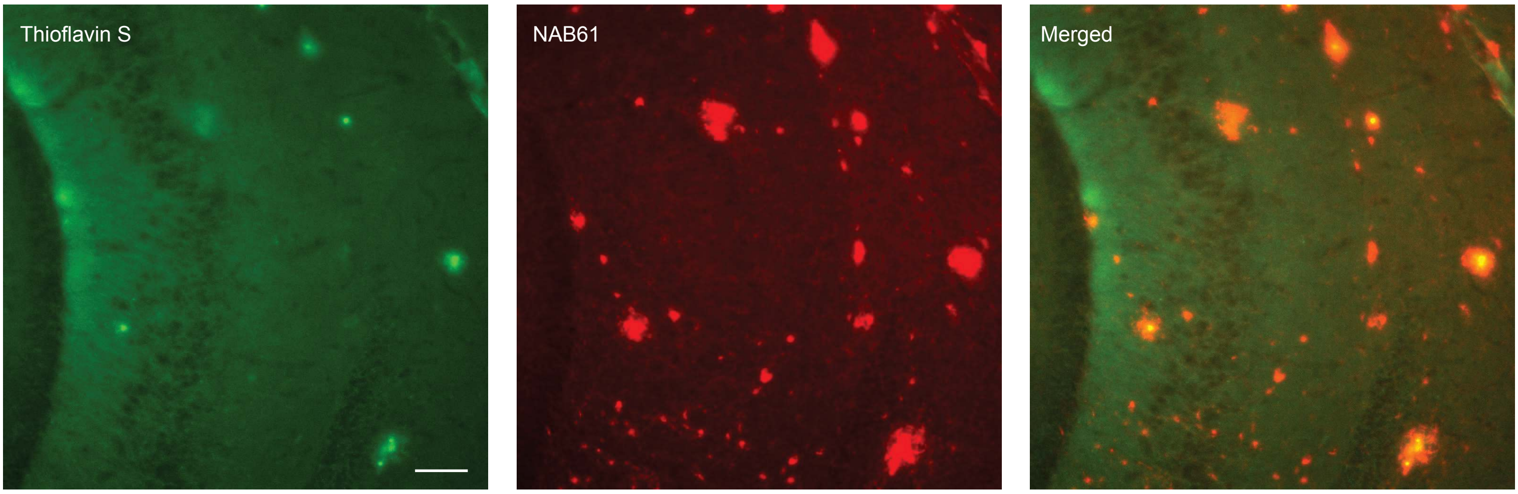

Figure S1 oAβ is the predominant Aβ species, instead of Aβ plaques in CA3 of 3-4 mo APP$^{NL\text{-}G\text{-}F}$ mice. Representative images of Thioflavin S (green) and NAB61 (red) staining in hippocampal brain slices. Scale bar: 100 µm. Thioflavin S is used to stain amyloid plaques. NAB61 (gift of V. Lee, University of Pennsylvania, PA) is oAβ-specific antibody. The co-staining method was performed as described by Liu et al., J Med Chem, 2024.
