# Supplementary material for: Inhibition of hippocampal mossy fiber plasticity and episodic memory by human Aβ oligomers is prevented by enhancing cAMP signaling in Alzheimer's mice
Source: Alzheimers Dement. 2025 Apr 29;21(4):e70194. doi: 10.1002/alz.70194 (PMC12040739; doi:10.1002/alz.70194)
Supplement: Supplementary file 2 — Supporting Information [file ALZ-21-e70194-s004.pdf]

# FIGURE S2

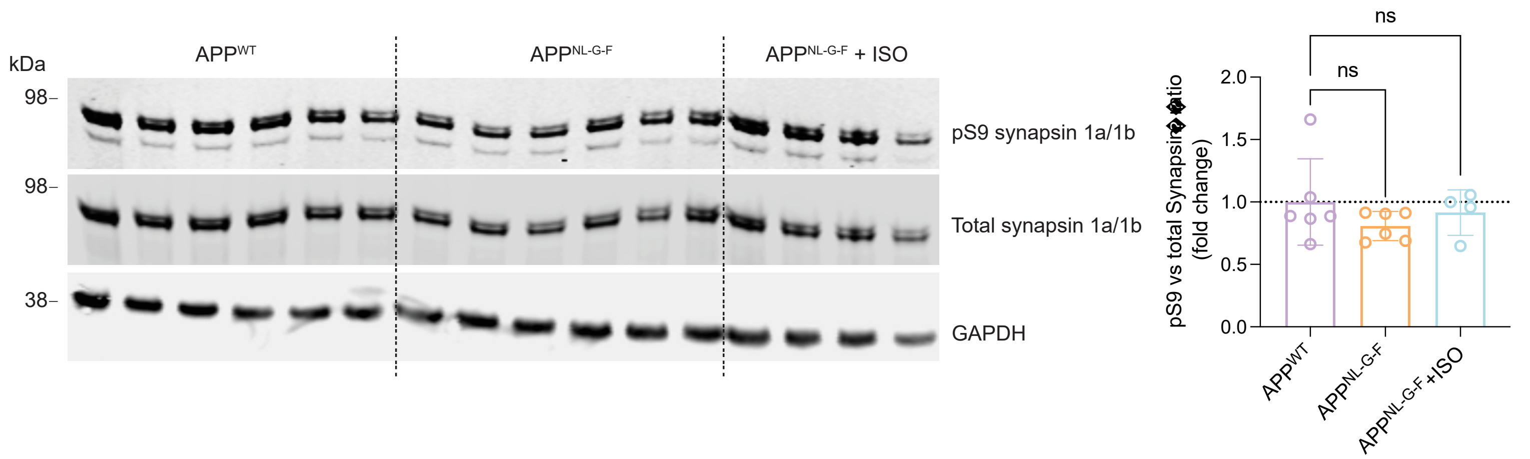


**Figure S2 No significant change in pS9 to total synapsin I ratio in 3-4-mo APPNL-G-F mice.** Western blots for total and pS9 synapsin I. GAPDH is a loading control. Mean +/- SD; ns, not significant. Brown-Forsythe and Welch ANOVA with Dunnett's T3 post hoc test for multiple comparisons.
