# Supplementary material for: Inhibition of hippocampal mossy fiber plasticity and episodic memory by human Aβ oligomers is prevented by enhancing cAMP signaling in Alzheimer's mice
Source: Alzheimers Dement. 2025 Apr 29;21(4):e70194. doi: 10.1002/alz.70194 (PMC12040739; doi:10.1002/alz.70194)
Supplement: Supplementary file 3 — Supporting Information [file ALZ-21-e70194-s002.pdf]

FIGURE S3

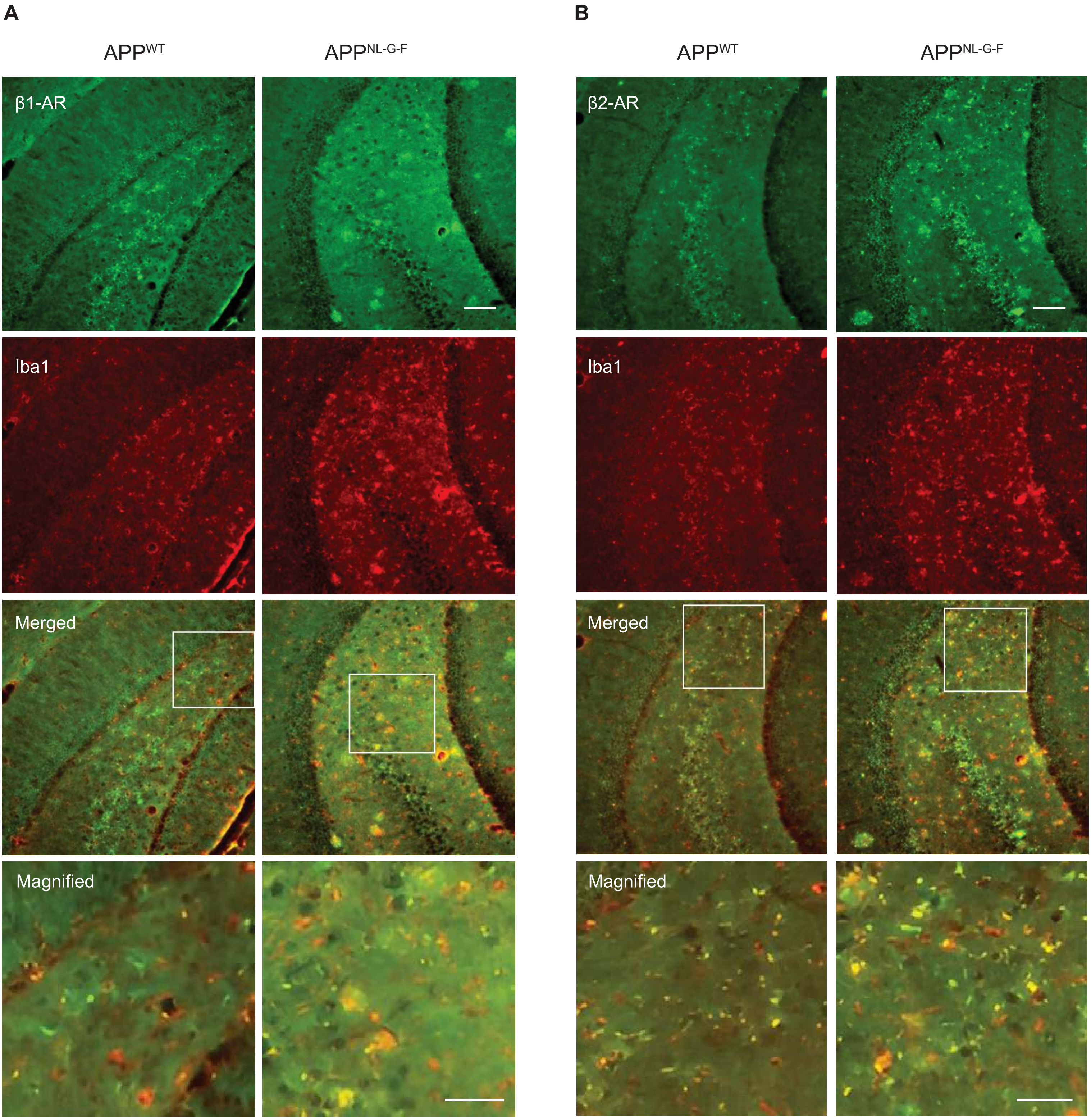

Figure S3 β1-AR and β2-AR are co-localized with microglia in CA3 of 6-7 mo wt and APP^{NL-G-F} mice, respectively. Double immunofluorescence for β1-AR and Iba1 (A) and β2-AR and Iba1 (B). Scale bar: 100 μm for whole sections and 50 μm for magnified sections from white box in panel A and B.
