# Supplementary material for: Inhibition of hippocampal mossy fiber plasticity and episodic memory by human Aβ oligomers is prevented by enhancing cAMP signaling in Alzheimer's mice
Source: Alzheimers Dement. 2025 Apr 29;21(4):e70194. doi: 10.1002/alz.70194 (PMC12040739; doi:10.1002/alz.70194)
Supplement: Supplementary file 4 — Supporting Information [file ALZ-21-e70194-s003.pdf]

FIGURE S4

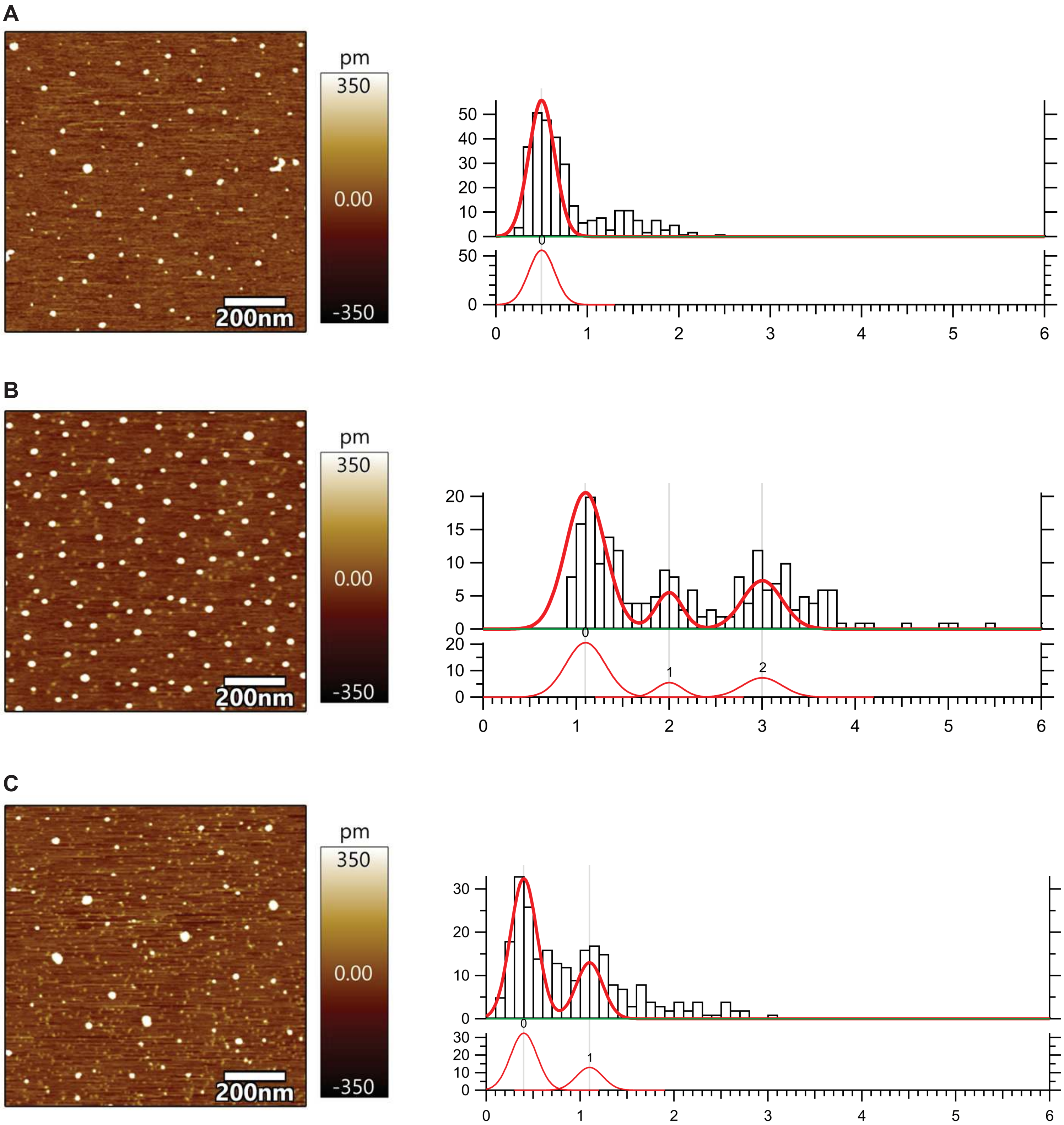

Figure S4 Typical AFM topographic images and the corresponding contrast height histograms of Aβ forms. A freshly prepared solution of monomeric synthetic Aβ42 (A) with single Gaussian peak at 0.6 ± 0.18 nm (n = 4 aliquots) was incubated for 3-6 hr at 4 °C to form Low-n Aβ42 oligomers (B): three Gaussian peaks at 0.98 ± 0.16, 1.8 ± 0.32 and 2.38 ± 0.41 nm (n = 5 aliquots). (C): two Gaussian peak at 0.66 ± 0.13 and 1.18 ± 0.07 nm (n = 3 aliquots) for pure synthetic Aβ37 monomers incubated for 3-6 hr at 4 °C.
