# Supplementary material for: Inhibition of hippocampal mossy fiber plasticity and episodic memory by human Aβ oligomers is prevented by enhancing cAMP signaling in Alzheimer's mice
Source: Alzheimers Dement. 2025 Apr 29;21(4):e70194. doi: 10.1002/alz.70194 (PMC12040739; doi:10.1002/alz.70194)
Supplement: Supplementary file 5 — Supporting Information [file ALZ-21-e70194-s001.pdf]

FIGURE S5

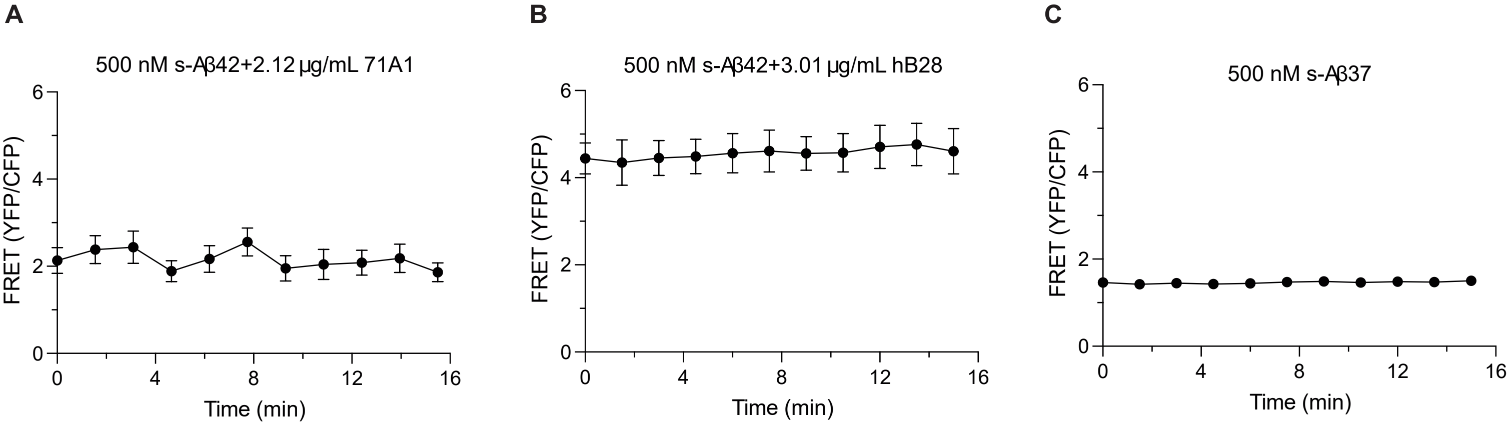

Figure S5 Negative controls for s-Aβ42 do not change FRET signal in DG granule cells. Neutralization of oAβ42 by soluble oligomer-preferring Aβ antibodies, 71A1 (2.12 µg/mL) or hB28 (3.01 µg/mL) prevents alteration of the FRET signal in the DG granule cells (A, B); n > 10 neurons for each group. (C) 500 nM synthetic Aβ37 monomers do not change the FRET signal in the DG granule cells; n > 10 neurons.
